# Electron scattering properties of biological macromolecules and their use for cryo-EM map sharpening†

**DOI:** 10.1039/d2fd00078d

**Published:** 2022-05-12

**Authors:** Alok Bharadwaj, Arjen J. Jakobi

**Affiliations:** a Department of Bionanoscience, Kavli Institute of Nanoscience, Delft University of Technology The Netherlands a.jakobi@tudelft.nl

## Abstract

Resolution-dependent loss of contrast in cryo-EM maps may obscure features at high resolution that are critical for map interpretation. Post-processing of cryo-EM maps can improve the interpretability by adjusting the resolution-dependence of structure factor amplitudes through map sharpening. Traditionally this has been done by rescaling the relative contribution of low and high-resolution frequencies globally. More recently, the realisation that molecular motion and heterogeneity cause non-uniformity of resolution throughout the map has inspired the development of techniques that optimise sharpening locally. We previously developed *LocScale*, a method that utilises the radial structure factor from a refined atomic model as a restraint for local map sharpening. While this method has proved beneficial for the interpretation of cryo-EM maps, the dependence on the availability of (partial) model information limits its general applicability. Here, we review the basic assumptions of resolution-dependent contrast loss in cryo-EM maps and propose a route towards a robust alternative for local map sharpening that utilises information on expected scattering properties of biological macromolecules, but requires no detailed knowledge of the underlying molecular structure. We examine remaining challenges for implementation and discuss possible applications.

## Abstract 2

We explore the use of generalised scattering properties of biomolecules for restoration of contrast and visual improvement of cryo-EM maps.

## Graphical abstract

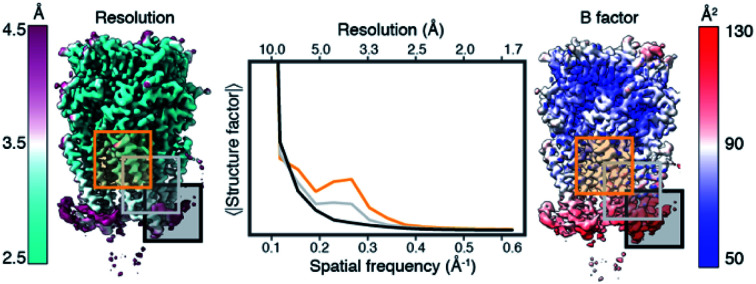

## Introduction

Cryogenic electron microscopy (cryo-EM) enables structure determination of biological macromolecules and their assemblies. Technological advancements have transformed single-particle cryo-EM into a central tool for structural biology that now routinely produces high-resolution structures of proteins and protein complexes, approaching atomic resolution in favourable cases.^1,2^ Three-dimensional single-particle cryo-EM maps are obtained by combining information from many images of a macromolecule in differing orientations, where each image is a projection along the direction of the incident electron beam. The low dose that can be tolerated by biological samples before damage occurs leads to imperfect images with very low signal-to-noise ratio.^3,4^ The sample will typically represent an ensemble of the molecules and assemblies in different conformations and compositions. The requirement to average low signal-to-noise ratio images of structurally different samples to generate a cryo-EM reconstruction implies that the resulting three-dimensional structure will be an average representation of the input conformers or assemblies. Together with noise from solvent scattering, optical aberrations and inaccuracies in the particle alignment process, such sample heterogeneity contributes to systematic variation of the signal-to-noise ratios in different regions of the reconstructed electrostatic potential map. Regions that are structurally very similar across many particles will produce a stronger signal than regions that are different in structure or molecular composition.^5,6^

The signal-to-noise ratio in the images used in reconstruction and in the resulting electrostatic potential maps is highly resolution-dependent. This resolution dependence is typically captured in a B factor. The B factor models the collective effect of factors related to the sample, the experiment, and the data processing, as a Gaussian dampening function in reciprocal space.^7,8^ It is standard practice to estimate the B factor from a Fourier representation of the reconstructed volume and use this information to optimise the resolution-dependent weighting of its amplitudes to produce maps with increased clarity.^7,9–12^ Alternative scaling procedures have also been proposed.^13–16^ The desired outcome of all procedures is to enhance map contrast such that it improves the representation of expected molecular features at a given resolution, and facilitates their interpretation in terms of atomic models.

Any filtering operation in Fourier space will have a global effect in real space and hence will affect all local regions equally. Because the signal-to-noise ratio typically varies between regions, global filtering is often inappropriate and can result in excessive blurring of high-resolution regions or oversharpening of noisy low-resolution regions of the map. This has lead to the development of methods that attempt to account for this variation by performing filtering locally.^17–22^ Several of these approaches are restrained by information that comes from expectations around general properties of macromolecular structures, and on their features in the real-space map or its Fourier space representation.^16–18,21^ We here review the spectral properties of biological macromolecules imaged by cryo-EM and explore how expectations around their scattering behaviour can be used to improve cryo-EM density maps.

## Results

### Fourier spectra of biological macromolecules

**Fig. 1:**
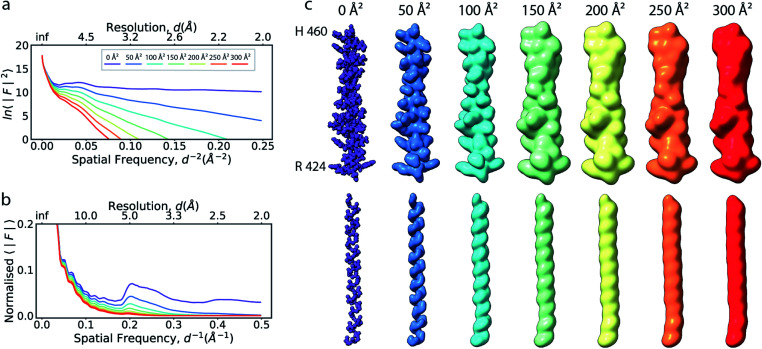
The effect of the B factor on radial amplitude spectra and appearance of real-space densities from biological macromolecules. (a) The natural logarithm of the radially averaged squared structure factors computed from the Fourier transform of an idealised α-helix (R^424^DWLRVGYVLDRLLFRIYLLAVLAYSITLVTLWSIWH^460^; PDB ID 6y5a), simulated at different overall B factors. (b) Radial profiles of the normalised structure factor computed for the helices. Note the decay of the distinctive local maximum at ∼4.5 Å with increasing B factor. (c) Real-space densities of the idealised α-helix (top) and its main chain trace (bottom) simulated at different B factors. The colour code for B factors is equivalent in (a–c).

The ideal three-dimensional reconstruction of a biological macromolecule or macromolecular complex would be a map of its electrostatic potential at every point on a three-dimensional grid. The signal in an electron potential map fades with resolution, and as a consequence structural features towards the resolution limit may become increasingly weak and difficult to interpret. This behaviour can be readily visualised by representing a map as a Fourier series and analysing the resolution dependence of the signal in the reconstruction by spherically averaging over the amplitudes |*F*_observed_(*s*)| of its Fourier coefficients *F*_observed_(*s*) – also called structure factors – at each spatial frequency *s*. The result of this procedure is a one-dimensional spectrum (or radial profile) that contains information of the average image amplitude as a function of resolution. For biological macromolecules, this amplitude spectrum can be thought of as a representation of protein texture in reciprocal space and yields a profile with highly predictable features (Fig. 1a and b): the spectrum peaks at the origin, where all atoms scatter in phase and its amplitude is proportional to the number of scattering atoms in the reconstructed volume. At frequencies below ∼0.1 Å^−1^ (0.01 Å^−2^ in Fig. 1a), the spectrum displays an approximately quadratic decay characterised by the shape of the molecular envelope and solvent. Beyond a resolution of ∼12–8 Å (see Singer (2021) for an accurate derivation^24^), the spectral behaviour can be approximated by that of a uniformly random collection of independent atoms, and its average amplitude is equivalent to the sum of the squared atomic form factors:^25^
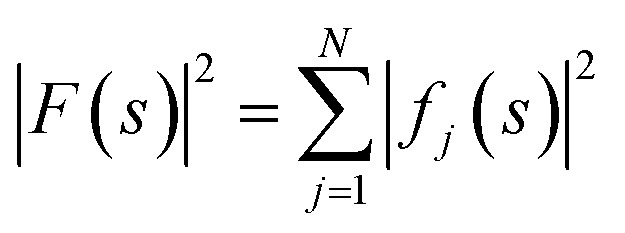


The frequency range over which this relationship is applicable is referred to as the Wilson range. The regular, recurring three-dimensional arrangement of adjacent amino acid residues in protein secondary structures, or bases in nucleic acids, introduces characteristic features in the radial pair-distribution function that deviate from the assumption of a uniformly random distribution. This results in amplification or dampening of structure factor amplitudes at certain frequencies in the Wilson range, leading to prominent peaks or dips in the radial profile.^26^

If the atoms were ideal point scatterers, the overall structure factor would be constant beyond the cutoff frequency of the applicable Wilson range, leading to a “flat” spectrum. Because atoms have shape and their form factors fall off smoothly with increasing frequency, the overall structure factor amplitude also decays smoothly as a function of resolution. In addition, the attenuation of amplitudes as a function of spatial frequency is dependent on a B factor. The B factor models the collective effects that result in frequency-dependent dampening of signal in reciprocal space.^7^ Higher values indicate stronger dampening and therefore lower signal at higher resolution (Fig. 1a and b). The effect of this behaviour in real space is the progressive loss of high-resolution detail as the B factor increases (Fig. 1c and S1†). The dampening envelope also affects the deviations caused by secondary structure from the expected fall-off in the Wilson range, which become increasingly obscured with increasing B factor (Fig. 1b). In real-space density maps this manifests as the progressive blurring of discernible periodicities in secondary structure elements (Fig. 1c). If the overall B factor is known, the original contrast can be restored by multiplying each Fourier coefficient with the inverse of the dampening function:*F*_restored_(*s*) = *F*_observed_(*s*)e^−(*B*_sharpen_*s*^2^/4)^,where *F*_restored_(*s*) and *F*_observed_(*s*) are the contrast-enhanced and observed Fourier coefficient at frequency *s*, and *B*_sharpen_ = −*B*_overall_. This process is known as map sharpening.

### Robustness of local B factor estimation in cryo-EM maps

**Fig. 2:**
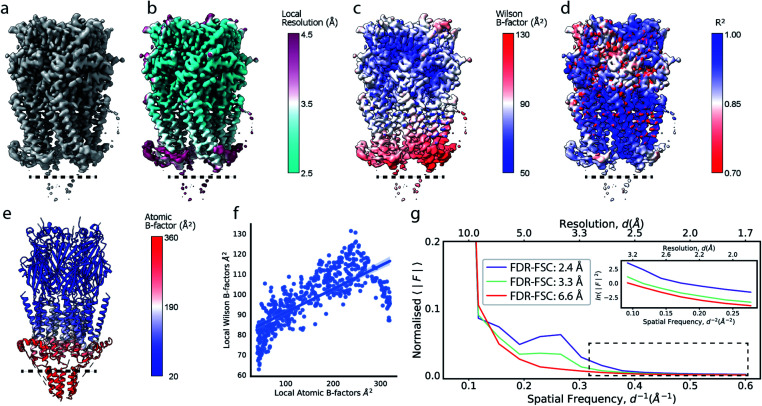
Local resolution and B factor variation in cryo-EM reconstructions. (a) Serotonin-bound 5-HT_3A_ receptor (EMD-10692; thr. 0.015). (b) Local resolution mapped onto the surface representation of EMD-10692. (c) Local B factors, and (d) the square of the sample correlation coefficient of the linear regression (coefficient of determination, *R*^2^) from fitting to the Wilson range, mapped onto the molecular surface of EMD-10692. The dashed line separates a poorly resolved region. (e) Cartoon representation of a re-refined atomic model of 5-HT_3A_ receptor (PDB ID 6Y5A) coloured by residue-averaged atomic B factor. (f) Correlation between local B factors estimated by Wilson fitting and average B factor from the atomic model. (g) Radial amplitude profiles for 5-HT_3A_ receptor (EMD-10692) for map regions at high, intermediate and low resolution as determined by the FDR-FSC procedure.^23^ The insert shows an close-up of the region indicated by the dashed box.

Cryo-EM reconstructions frequently display substantial variation in local resolution (Fig. 2a and b). These arise, for example, from non-coherent averaging due to structural flexibility inherent to macromolecules, or from differences in molecular composition of the averaged macromolecular assemblies. In addition to effects from conformational and compositional heterogeneity, the quality of three-dimensional reconstructions is typically poorer at the particle periphery when compared with the particle center due to errors in orientation assignment and limitations of current reconstruction algorithms. All of these effects result in lower resolution in specific regions affected by these factors. Analogous to estimating local resolution through convolving each voxel in the reconstruction with a mask (windowing) and calculating the Fourier Shell Correlation (FSC)^27,28^ between two equivalent windowed volumes from two independent half maps,^5^ local B factors can be estimated by line fitting to the Wilson range of radial profiles calculated from the Fourier transform of the windowed volumes. The slope of this line is referred to as the Wilson B factor. Local resolution is correlated with local B factors;^17^ density regions with low local resolution display high local B factor, and *vice versa* (Fig. 2c). It should be noted that determination of local B factors by linear fitting to Guinier profiles from windowed volumes is frequently not robust, as reflected by the coefficients of determination *R*^2^ of local B factor estimates (Fig. 2d). First, the number of available samples available is reduced in smaller, windowed regions of the map and local fitting is inherently more noisy and variable. Perhaps counter-intuitively, the quality of fit can also be adversely affected in areas of higher resolution that extend only little further than 3 Å, where secondary structure modulations in the Wilson range of the radial amplitude profile are most prominent (Fig. S2a–c†). In cases where map resolution extends sufficiently far (∼2.5 Å and beyond), line fitting can be done over the resolution range unaffected by these modulations.

Alternatively, and perhaps more robustly, local B factors can be estimated if atomic models are available for which atomic displacement parameters (ADPs)^29,30^ can be refined (Fig. 2e and f). ADPs are related to the mean square displacement of atoms and describe their degree of static and dynamic disorder, and errors that may exist in the model. Atomic B factors can be computed from ADPs by the expression:*B*_*j*_ = 8π^2^〈*u*_*j*_^2^〉,where 〈*u*^2^〉 is the isotropic ADP that represents the mean square displacement from the mean position of the atom *j*. Any uncertainty in the atomic position blurs the contribution of that atom to the density. It follows that variation of ADPs changes the atomic contributions to the overall, or local, calculated structure factor:
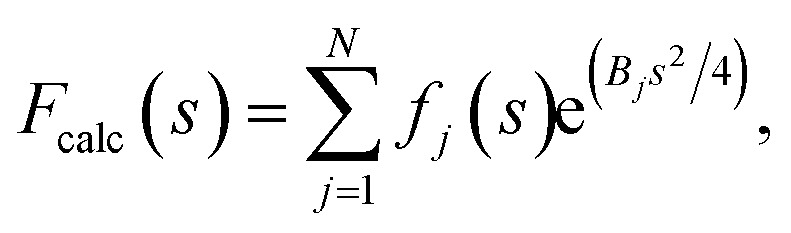
where *f*_*j*_ is the spherically symmetric form factor of atom *j* and *B*_*j*_ its isotropic atomic B factor. By optimising the correspondence between calculated and observed structure factors (or real-space densities), atomic B factors can be determined during atomic model refinement.^30–32^ Since bonded atoms cannot oscillate independently, atomic models provide the opportunity to restrain refinement of ADPs to follow reasonable physical assumptions such that refined ADPs within residues, within a sequence of residues or within rigid domains are locally correlated.^31–34^ We here assume atomic B factors to be isotropic, while in reality the extent of blurring can be dependent on direction, for example due to relative mobility of different domains and/or uncertainty in the particle alignments. Such dependencies could be accounted for if robust protocols for anisotropic refinement of B factors, such as TLS refinement,^35,36^ can be established for cryo-EM data.

If estimated reliably, local B factors can serve as an effective means to optimise local contrast in cryo-EM density maps. Since the B factor determines the decay rate of the frequency-dependent amplitude fall-off, local scaling of experimental map amplitudes using radial profiles computed from a B factor-weighted reference structure represents a form of local signal-to-noise weighting (Fig. 2g). These properties have been exploited previously for local map sharpening in the program *LocScale*.^17^

### Reference-based local sharpening of cryo-EM maps

**Fig. 3:**
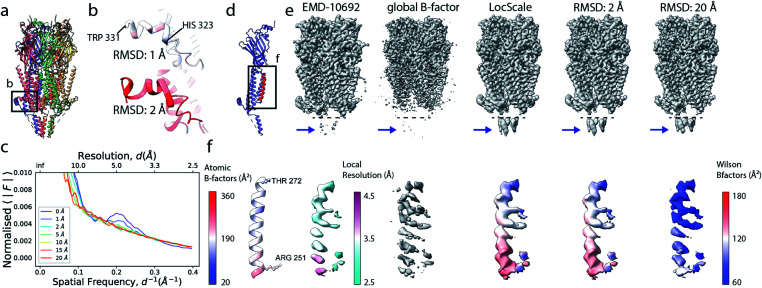
The effect of model accuracy on reference-based local sharpening. (a) Cartoon representation of the unperturbed atomic model of 5-HT_3A_ receptor (PDB ID 6Y5A). (b) Close-up of a specific region of perturbed atomic models, the displayed region in boxed out in (a). (c) Radial profiles of electrostatic potential maps computed from perturbed models. (d) Cartoon representation of a single chain of 5-HT_3A_ highlighting (red) the helical region shown in (f). (e) Unprocessed map of 5-HT_3A_ receptor (left; EMDB-10692) and globally or locally sharpened maps obtained from unperturbed or coordinate-perturbed atomic models. (f) Close-up of scaled density of helix Arg^251^-Thr^272^ from chain A of PDB 6Y5A coloured by local B factor. Also shown is the cartoon representation of the helix segment (left) coloured by residue-averaged B factor.

Local sharpening using current implementations of *LocScale* is implicitly dependent on the availability of an atomic model. At the same time, the sharpening procedure itself serves to facilitate the interpretation of the experimental map in terms of such a model, which seems contradictory. Fortunately, in many cases at least partial model information will be available and we have shown previously that this often is a sufficiently good starting point for local sharpening using an atomic model ref. 17. To quantify the accuracy of the prior information on the molecular structure required to perform effective sharpening using reference-based scaling, we performed a series of experiments using a reconstruction of 5-hydroxytryptamine receptor 3A (5-HT_3A_) bound to 5-HT (serotonin)^37^ as our model system. In a first experiment, we randomly perturbed atom positions of the atomic model at a root mean square deviation (RMSD) of 1–20 Å within a mask encompassing the molecular envelope and computed reference maps from the perturbed models to scale the experimental density using *LocScale* (Fig. 3a and b). We then compared the resulting maps to the LocScale map computed from the original atomic model by computing their pairwise FSCs. While even at 20 Å RMSD the overall effect is relatively small, increasing levels of perturbation lead to gradual lowering of the FSC, most strongly affecting frequency ranges close to the maximum map resolution (Fig. S3a†). At the same time, increasing RMSD affects the relative strength of the secondary structure deviations in the radial profile (Fig. 3c). Perturbation of the underlying atomic model results in two alterations of the reference structure that can affect the effectiveness of reference-based local sharpening. First, the random displacement of atoms changes the underlying molecular structure, and hence the radial structure factor of a windowed volume, which will progressively break the spatial periodicity of secondary structure elements and approximate a true Wilson distribution of uniformly random, independent atoms. Our results show that the deviations of the expected fall-off in the Wilson range persist to ∼2 Å random error, while above this value secondary structure becomes hard to assign as these modulations have essentially vanished (Fig. 3b and c). Second, the perturbation of atomic positions will also increasingly change the local B factor distribution for structures that display significant variation in local B factors. Effectively, as the model perturbation increases, local B factor correlations are gradually lost until converging to a uniform average B factor across the volume contained within the mask.

**Fig. 4:**
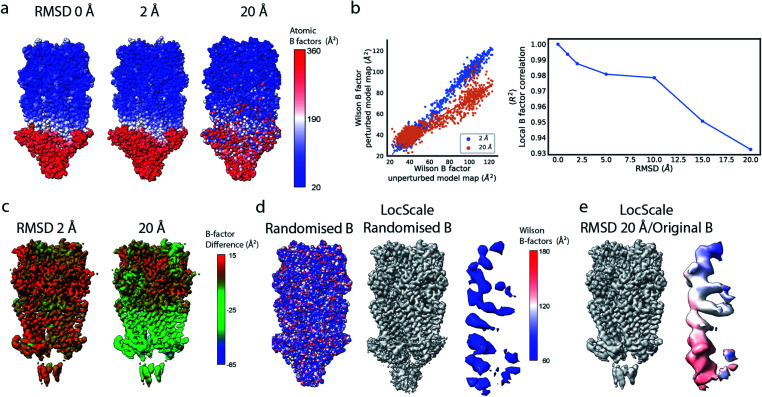
Effect of local B factor estimation on reference-based local sharpening. (a) All-atom representations of perturbed atomic models. Atoms are shown as spheres and coloured by atomic B factor. (b) Local B factor correlation plot for unperturbed and perturbed models (left) and dependence of the coefficient of determination (*R*^2^) on RMSD to the unperturbed structure (right). (c) Distribution of local B factor differences Δ*B* mapped onto the molecular surface of 5-HT_3A_ receptor. (d) All-atom representation of the original model with randomised B factor (left) and LocScale map obtained using the randomised B factor reference. (e) LocScale map obtained using the 20 Å RMSD-perturbed atomic model with original B factor.

Compared to uniform sharpening with a global average B factor and consistent with previous observations,^17^ local reference scaling using an optimal model improves the representation of density features by locally balanced sharpening and blurring in regions deviating from the average map resolution (Fig. 3e). Closer inspection of three representative regions encompassing helices M2 and MX, as well as the 5-hydroxytryptamine ligand, reveals the effect of reference perturbation on the quality of local sharpening (Fig. 3f and S3c–e†). Consistent with the observed effect on the radial profile (Fig. 3c), perturbations <5 Å RMSD have relatively little effect on the average local B factor and the result of local sharpening. Only at RMSD >10 Å is the local B factor distribution considerably altered and begins to affect the accuracy of local scaling (Fig. 4a–c, S3c and S4b, c†), ultimately approaching that of global sharpening (Fig. 4d). In contrast, models with coordinate errors as large as 20 Å show adequate scaling if local B factors are estimated correctly (Fig. 4e). This indicates that even initial models with significant errors can serve as meaningful scaling references provided their ADPs can been refined.

### Reference-based local sharpening using average profiles

**Fig. 5:**
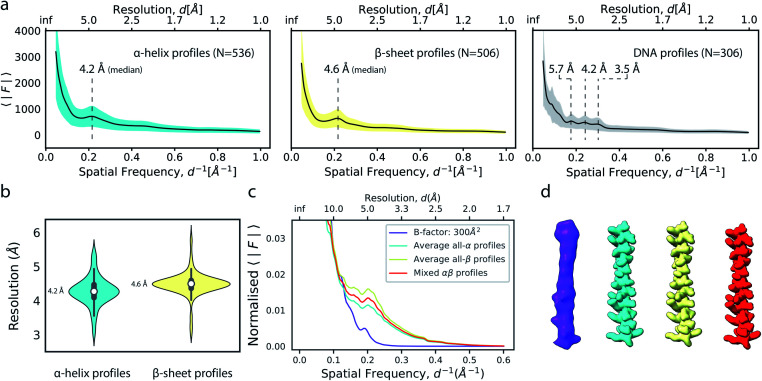
Modulation of the radial amplitude profile for proteins and nucleic acids. (a) Average radial amplitude profiles for α-helical, β-sheet and DNA structures. Solid lines and shaded regions represent mean and ±1.0*σ* confidence intervals, respectively. (b) Violin plots of the major maximum in the Wilson range of α-helical and β-sheet profiles. (c) Radial amplitude profiles and (d) real-space densities for an idealised α-helix (R^424^-H^460^; PDB ID 6y5a) obtained by scaling a blurred map (*B* = 300 Å^2^) with average reference profiles from all-α, all-β or mixed-type profiles.

We next explored the variation of secondary structure modulations in the Wilson range of radial profiles computed from a large set of experimental structures. From a set of 1000 structures randomly selected from the Protein Data Bank (PDB),^38^ we extracted helix, sheet, DNA and RNA residues and computed average radial profiles from simulated electrostatic potential maps. As we were interested only in the effect of secondary structure on the average profiles, waters, ligands, post-translational and base modifications as well as hydrogen atoms were excluded from these calculations. The folding of polypeptide chains into a three-dimensional structure gives rise to the canonical α-helix and β-sheet elements of protein secondary structure. These features produce characteristic distances within the folded protein chain that range between 4.5–7 Å. Our analysis revealed that experimental profiles of secondary structure elements gave rise to highly reproducible peaks and dips of the profiles within the Wilson frequency range. We identified a strong local minimum at ∼6.2 Å (α,β), as well as characteristic peaks at 4.2 Å (α), 4.6 Å (β) and in the 3–6 Å range (DNA) (Fig. 5a), consistent with expectations from the pair distribution function of these structural elements.^26,39^ While the average profiles for α and β appear very similar, their distributions and the position of the local maximum of the modulations in the Wilson range are distinctly different (Fig. 5b). We did not find characteristic fingerprints in RNA profiles, possibly because of the large variation in RNA secondary structure (Fig. S5a†). The overall highly similar radial profiles suggest that generalised average profiles could be used as reference profiles in cases where explicit model information is missing. We performed a series of experiments to test this possibility. First, we computed a ground truth electrostatic potential map for an α-helix encompassing residues Arg^424^-His^460^ of (5-HT_3A_) receptor with isotropic B factor of 50 Å^2^. We then blurred the map with an additional B factor of 250 Å^2^ to generate a target map for density scaling. Next, we scaled the target map by imposing the generalised reference profiles for α-helical and β-sheet texture, or a mixed αβ profile obtained from averaging α-helical and β-sheet profiles, each matched to the reference fall-off with a B factor of 50 Å^2^. LocScale sharpening using either of the three generalised profiles returned essentially identical sharpened maps with mutual real-space correlation coefficients RSCC >0.995 (Fig. 5c, d and S5b†). From the above, it follows that implementation of local sharpening could be achieved using reference profiles instead of explicit model information. It should be noted that, whereas average profiles in the Wilson range are very similar across many protein structures, they may differ substantially in the low resolution range where the fall-off is primarily dependent on molecular shape (Fig. S5c†).

### Limitations of local sharpening using model-based references

**Fig. 6:**
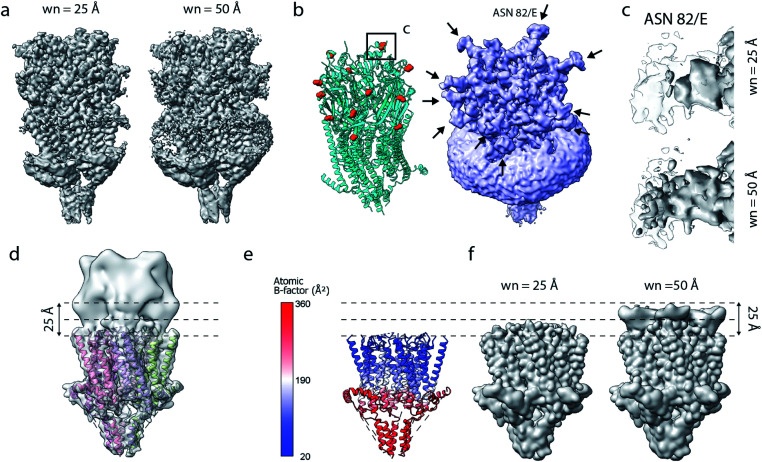
Limitations of model-based sharpening. (a) LocScale maps for 5-HT_3A_ (thr. 0.08) obtained using a window size of 25 Å (left) and 50 Å (right). (b) Fitted atomic model (PDB ID 6Y5A) with asparagine residues at N-linked glycosylation sites shown as spheres (left) and confidence map at 1% false discovery rate (FDR, right). (c) Detailed view of one of the glycosylation sites (Asn^82^, chain E) from the LocScale maps obtained with 25 Å (top) and 50 Å (bottom) scaling windows. The transparent outline shows the contour of the confidence map at 1% FDR. (d) Synthetic map of 5-HT_3A_ with the top part intentionally blurred, and (e) a partial model used as a scaling reference superimposed on (d). (f) LocScale maps obtained using a partial model with a window size of 25 Å (left) and 50 Å (right).

Current implementations of reference-based local density sharpening depend on the availability of at least approximate model information, which can give rise to important limitations.^17^ We here used 5-HT_3A_ as a model system to illustrate these issues. One example is the presence of post-translational modifications such as N-linked glycosylation, with often heavily branched glycan chains that may extend substantially beyond the protein surface.^40^ Due to their inherent flexibility and associated weak density, these modifications can often not be built reliably in globally-sharpened maps. Similar issues arise with detergent belts, or lipid discs, in reconstructions of purified membrane proteins (Fig. 6a and b). If the glycan is not, or only partially modelled, then a model-dependent scaling procedure may lead to inadvertent dampening of the weak glycan-associated signal outside the modelled molecular boundary. This is readily visible when comparing a locally sharpened map for EMD-10692 obtained with *LocScale* using a reference model not containing glycans, and a confidence map thresholded at 1% false discovery rate (FDR) (Fig. 6a). Confidence maps are based on the statistical framework of multiple hypothesis testing and provide a robust way to visualise “true” molecular boundaries of cryo-EM electrostatic potential maps irrespective of the relative strength of density signal.^41^ To appreciate the effect of the model-based reference on local sharpening, it is instructive to zoom in closer on one of the glycosylation sites in 5-HT_3A_ (Asn^83^). Whereas the glycan density is emphasised in the LocScale map, the confidence maps of the same region span a substantially expanded volume associated with this glycosylation site, suggesting that the locally sharpened map does not recover the associated signal completely (Fig. 6c). This is related to the dimensions of the scaling window (25 Å), which will lead to dampening of any signal extending further than half the window size beyond the limiting voxel of the scaling mask.

Inadvertent dampening of signal by reference-based scaling is a prevalent issue whenever model information is incomplete. To further illustrate this effect, we generated a target map from an atomic model of 5-HT_3A_ in which we intentionally blurred a part of the map to simulate a density region that cannot be modelled (Fig. 6d and e). Using a partial model as the scaling reference will improve density representation in regions covered by this model, but will make density “disappear” progressively beyond the molecular boundary of the model (Fig. 6f). While some of the described effects can be mitigated by the use of larger scaling windows, or iterative schemes of scaling and model extension,^17^ these experiments illustrate an important limitation of the current implementations of reference-based scaling methods that may spill over to other approaches using the results of these methods, for example when using such maps as training targets for density modification based on deep neural networks.^22^

## Conclusions

Cryo-EM map sharpening remains an essential step of cryo-EM structure determination to guide atomic model building and help map visualisation. It has become apparent that global approaches are often insufficient to reflect the variation of signal-to-noise ratio in different regions of a cryo-EM reconstruction. Methods that attempt to optimise map contrast locally have become reliable tools in this process.^17–22,42^ It is an important requirement that such methods preserve the expected physical properties for electrostatic potential maps of biological macromolecules.^8,13,15–17,24,25^

We here reviewed the potential and limitations of incorporating prior knowledge about scattering properties and macromolecular features from known protein structures. Information such as expected scattering mass, the definition of boundaries between macromolecule and solvent, and secondary structure content can all be effectively used to restrain the scaling of cryo-EM density maps.^17,18,21^ We have previously shown that such information can be efficiently utilised by using an existing atomic model as a scaling reference. The general concepts laid out in this paper suggest that the restraints of reference-based sharpening can be exploited in future implementations without making explicit reference to atomic models. Indeed, our results demonstrate that model information is not necessarily required to approach optimal contrast restoration through reference-based sharpening. The challenge lies in implementing robust procedures to estimate overall scattering mass, local B factors and local secondary structure directly from the raw three-dimensional reconstruction. Once known with sufficient accuracy, these ingredients are sufficient to construct effective radial profiles for local scaling of Fourier coefficients in the absence of any atomic model. Such approaches will be useful to mitigate the risk of systematic bias introduced by incomplete model information. More generally, the requirement for robust map sharpening methods can be expected to increase as high-resolution subtomogram averages from *in situ* studies^43,44^ and native purifications^45,46^ accumulate, for which conformational heterogeneity may turn out to be yet more prominent and the exact molecular composition possibly unknown. We are working towards such methods.

## Methods

All the data processing was done using Python. The latest implementation of *LocScale*^47^ was used to perform map sharpening.

Analysis and data processing was performed using our in-house Python library *EMmer*.^48^*EMmer* makes use of functions from mrcfile,^49^ gemmi^50^ and Biopython^51^ to perform computations related to 3D density maps and atomic coordinates. Numerical computation and statistical analysis was done using the numpy, scipy and scikit-learn Python libraries. The PWLF library was used to obtain a piece-wise linear fit function to extract information from radial profiles. Local resolution of the deposited half-maps was computed by permutation testing of FSC resolution using the FDR-FSC procedure.^23^

All graphical plots were made using seaborn and matplotlib libraries. All visualisation of maps and atomic coordinate models was performed using *UCSF ChimeraX* software (version 1.3).^52^

### Amplitude scaling

All the primary data analysed in this work are available in the public domain. The unsharpened and unfiltered cryo-EM density map of 5-HT_3A_ (EMD-10692) and the associated atomic coordinate model (PDB ID 6y5a) were obtained from the EMDataResource Project and the Protein Data Bank.^38^ The unprocessed map (the sum of the unfiltered half-maps) was uniformly sharpened with a B factor of −107 Å^2^ and used as a target for atomic model refinement. The deposited atomic model was re-refined using *REFMAC5* (ref. 31) with jelly body restraints.^49^ Atomic displacement parameters of the fitted atomic model were subsequently refined without positional refinement using *Servalcat*.^42^ Electron potential maps from the refined atomic model were generated using the pdb2map function in *EMmer*, which employs the gemmi.DensityCalculatorE class to calculate the B factor weighted structure factors using electron atomic form factors.^53^

*LocScale* performs amplitude scaling using a rolling window of 25 Å. For each local window, the radially averaged amplitudes of the unsharpened window are scaled to match the radial profile of the reference map window by multiplication with a frequency-dependent scaling factor *k*(*s*) ∈ ***R***_≥0_
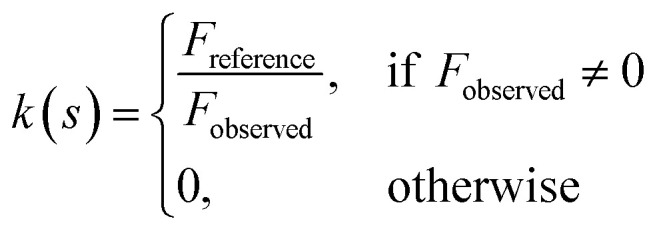
where *F*_reference_(*s*) ∈ ***R***_≥0_ and *F*_observed_(*s*) ∈ ***R***_≥0_ are radially averaged structure factor amplitudes at frequency *s* for the reference and the observed experimental map, respectively.

The value of the central voxel of the scaled window is then assigned to the corresponding voxel position of a new map. The process is repeated over all the voxels in order to obtain a contrast-optimised density map. To reduce computation time, the procedure was restricted to voxels contained in a binary mask, which was obtained from an atomic model (Fig. 3 and 4) or from an FDR based confidence map^41^ thresholded at 1% FDR (Fig. 6).

### Perturbation of reference models

All atom positions in the refined atomic model were perturbed randomly with an increasing RMSD magnitude. The position of each atom was perturbed as follows:***r***_*j*_ = ***r***^0^_*j*_ + ***r***^kick^

The magnitude of the kick vector is chosen randomly within a uniform distribution ranging between 0 and 2 × ***r***^kick^, where ***r***^kick^ is the desired RMSD (in Å). Atomic positions are perturbed such that the new positions lie within a defined molecular boundary to ensure that the total scattering mass per unit volume remains consistent for all structures. This mask is obtained by binarising a model map comprising all voxels extending 3 Å outward of atom positions in the model, and smoothing with a cosine edge filter over 5 voxels.

### Generation of average radial profiles

An initial set of 1000 coordinate models were randomly selected from the PDB. Among these, structures with a unit cell greater than 256 Å were discarded from analysis. The isotropic B factor of all atoms in the selected PDBs was set to zero. To obtain pure α-helix or β-sheet profiles for each structure, a secondary structure assignment was carried out for each residue using the DSSP algorithm.^54^ Each coordinate model was then split into three individual models based on the residue selection from the DSSP assignment, containing either α-helix, β-sheet or random coil residue sequences. Similarly, nucleic acid models were obtained by separating out residues containing the five canonical nucleobases for both DNA and RNA residues. Synthetic electrostatic potential maps for all models were calculated using the pdb2map function in the *EMmer* package. All synthetic maps were re-sampled on a cubic grid with a 256 Å cell edge and a voxel size of 0.5 Å. To avoid sampling errors during map generation from an atomic model with zero B factor, the set_refmac_compatible_blur method implemented in gemmi was employed to add an additional isotropic atomic displacement parameter *B*_add_ to compute the structure factors.^55^ Correction of this modification and extrapolation to zero B factor was then done by multiplying each Fourier coefficient with exp(*B*_add_*s*^2^/4) at each frequency *s*. Radially averaged Fourier amplitudes for each simulated density map were computed using the *EMmer* function compute_radial_profile. The resulting profiles for secondary structure and nucleotide types were averaged (α-helical, *N* = 536; β-sheet, *N* = 506; DNA, *N* = 306 and RNA, *N* = 247) to obtain reference average profiles and confidence intervals for each type.

To obtain the characteristic frequencies of Debye effects in all-α helix and all-β sheet profiles, a four segment piece-wise linear fit was performed using the PWLF library. The mean fit quality, *R*^2^, for the all-α helix profiles was found to be 0.98 (σ: 0.005) and that for all-β sheet profiles was 0.98 (σ: 0.007). Characteristic frequencies were extracted by filtering out the breakpoints between 6 Å and 3 Å.

## Author contributions

AB and AJ conceptualised the project. AB and AJ implemented the computational procedures and analysed results. AB and AJ wrote the paper. AJ supervised the project.

## Conflicts of interest

The authors have no conflicts to declare.

## Supplementary Material

FD-240-D2FD00078D-s001

## Data Availability

Cryo-EM density maps and models generated in this study are available at https://doi.org/10.5281/zenodo.6531395. Scripts used for analysis in this manuscript are available at https://gitlab.tudelft.nl/aj-lab/publications/.
