# Supplementary material for: Electron scattering properties of biological macromolecules and their use for cryo-EM map sharpening
Source: Faraday Discuss. 2022 May 12;240:168–83. doi: 10.1039/d2fd00078d (PMC9642005; doi:10.1039/d2fd00078d)
Supplement: FD-240-D2FD00078D-s001 [file FD-240-D2FD00078D-s001.pdf]

Electronic Supplementary Material (ESI) for Faraday Discussions.
This journal is © The Royal Society of Chemistry 2022

Electronic Supplementary Information

Exploiting physical properties of biological macromolecules for cryo-EM map sharpening

Alok Bharadwaj[a] and Arjen J. Jakobi[a*]
[a] Department of Bionanoscience, Kavli Institute of Nanoscience, Delft University of Technology, Delft, The Netherlands

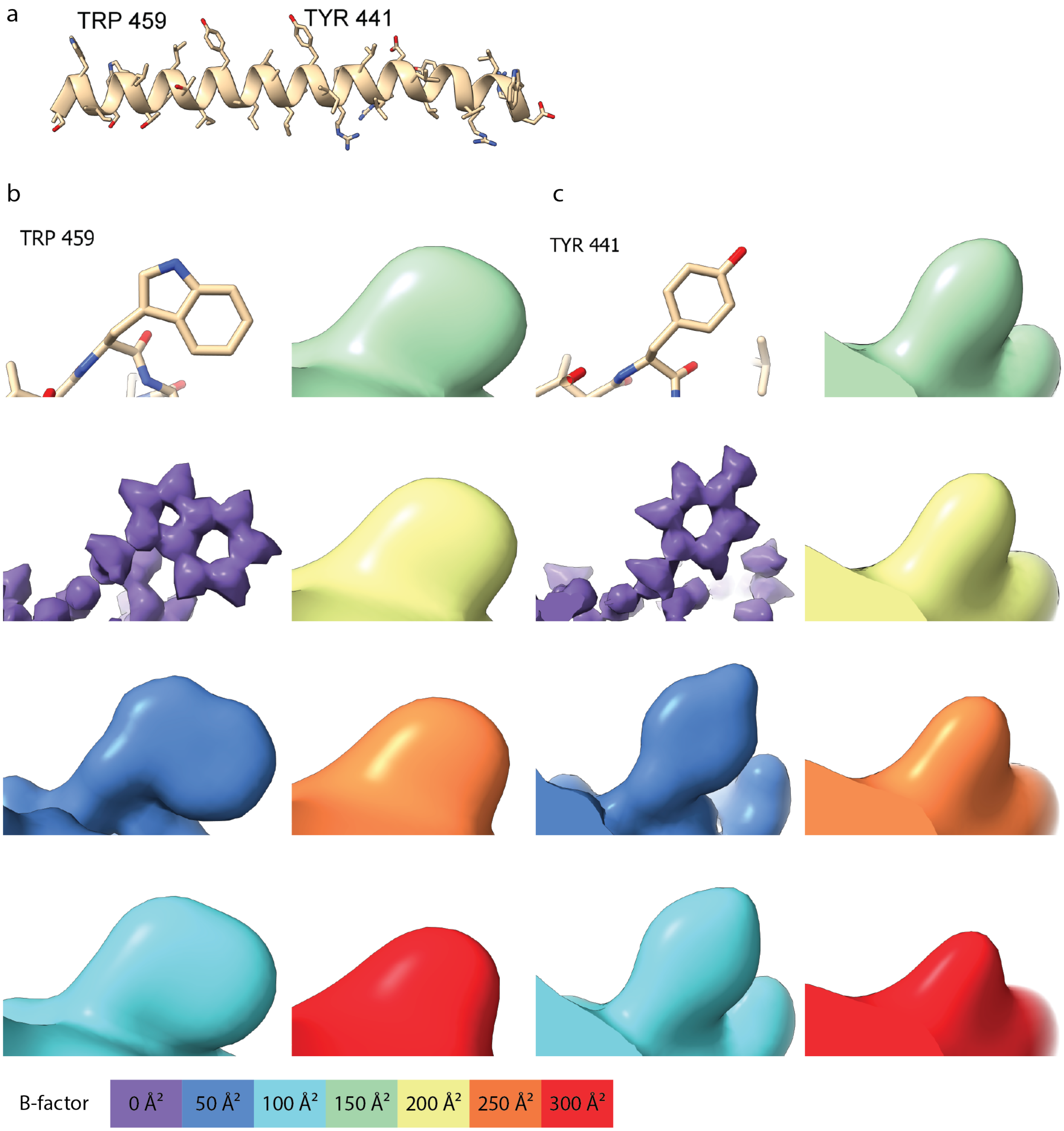

Fig. S1 Influence of B factor on real-space density of side chain representation. (a) Segment of α-helix(R^{424}DWLRVGYVLDRLLFRIYLLAVLAYSITLVTLWSIWH^{460}; PDB ID 6y5a) used to simulate real-space density at different atomic B factors. Close-up of side-chain density for (b) `TRP,459` and (c) `TYR,441` residues at different B factors. The colour codes for (b) and (c) follow the rainbow scheme shown at the bottom

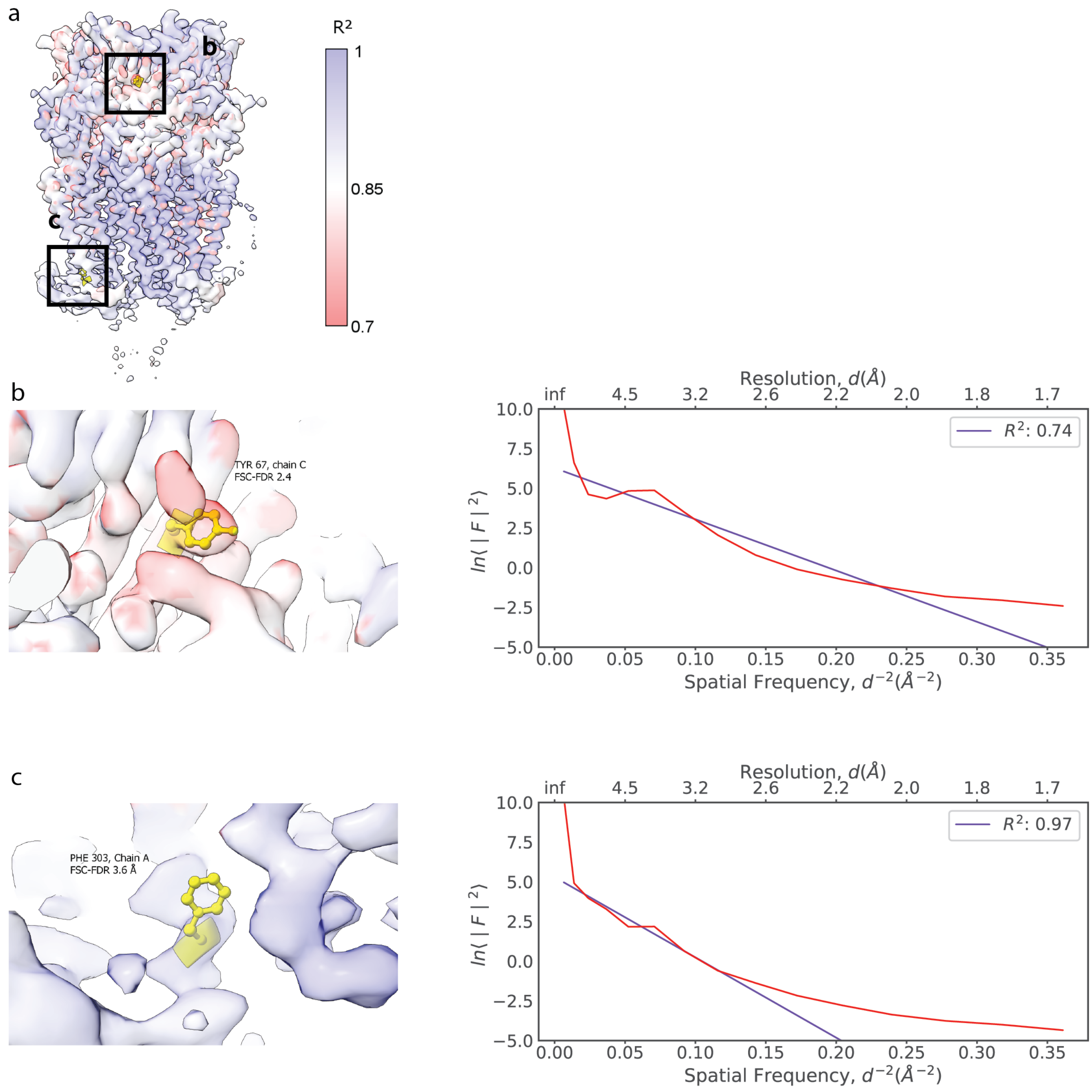

Fig. S2 Robustness of (local) B factor estimation by fitting in the Wilson region. (a) Coefficient of determination (R^2) from linear regression fitting in the Wilson regime mapped onto a surface representation of 5-HT3 (EMD-10692). (b,c) Real-space density segment (left) and radial profile (right) of two map regions with moderate (b) and good (c) coefficient of determination R^2 of the local B factor). Fitting in the better resolved region in (b; FDR-FSC:2.4 Å) is adversely affected by stronger secondary structure modulation of the amplitude spectrum.

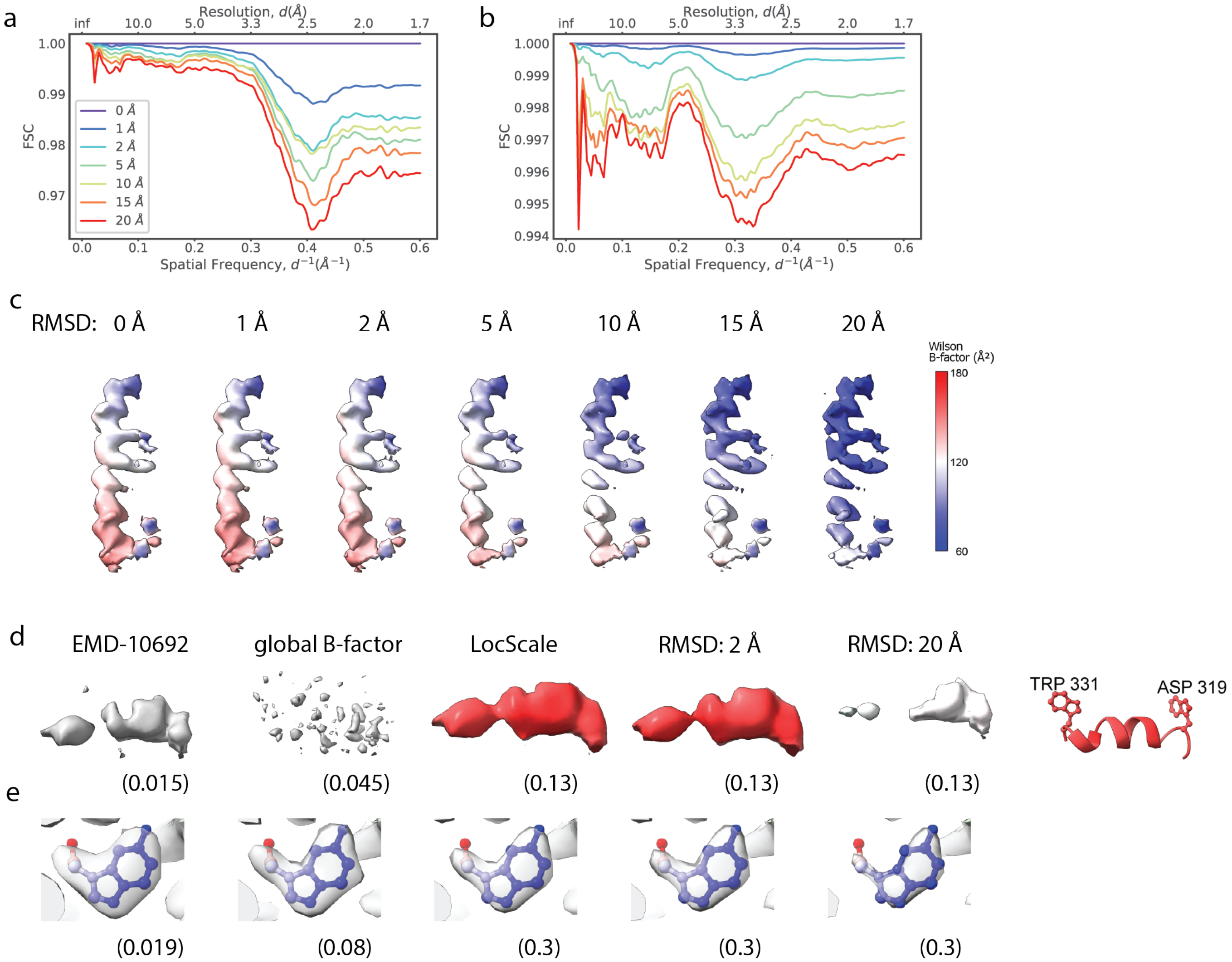

Fig. S3 Influence of model perturbation on reference-based scaling. (a) Fourier Shell Correlation of LocScale maps obtained using atomic models with increasing coordinate perturbation with respect to a LocScale map obtained with unperturbed atomic model (RMSD=0Å). Note that the perturbed curves show a dip near the location of maximum local resolution in the map ($\sim$ 2.5Å) (b) FSC dip analysis with an additional blur (B=100 Å^2) for the atomic models in (a). The position of dip in the FSC curves shifts left towards lower resolution when compared with (a). (c) Close-up of scaled density of helix Arg^{251}-Thr^{272} from chain A of PDB 6Y5A (coloured by local B factor) obtained using perturbed atomic models. (d) Close-up of helix Asp^{319}-Trp^{331} from chain E of PDB 6Y5A from the unprocessed map (left), globally sharpened map and LocScale maps obtained from unperturbed and perturbed atomic models. (e) Close-up of density enclosing the 5-hydroxytryptamine (serotonine) ligand in the unprocessed map (left), globally sharpened map and LocScale maps using unperturbed and perturbed atomic models. The threshold for each detail view is shown below its corresponding figure.

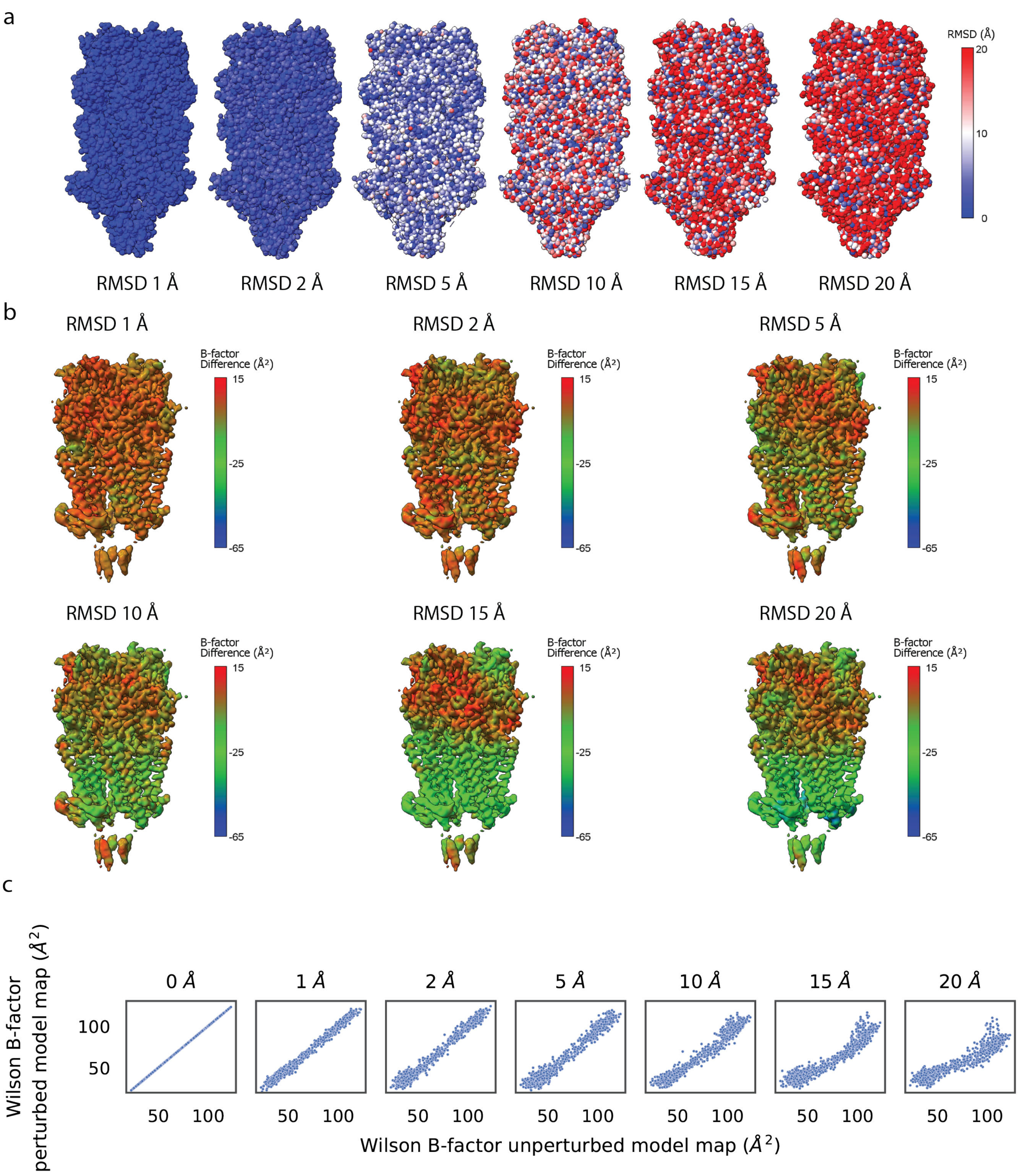

Fig. S4 Effect of model perturbation on B factor distributions. (a) All-atom representation of atomic models perturbed with an increasing RMSD magnitude coloured by the atomic displacement with respect to the unperturbed structure (b) Distribution of local B factor differences ΔB of the perturbed vs. unperturbed models mapped onto the surface representation of the LocScale map obtained from an unperturbed atomic model. (c) Local B factor correlation plots for the unperturbed and perturbed models for different RMSDs.

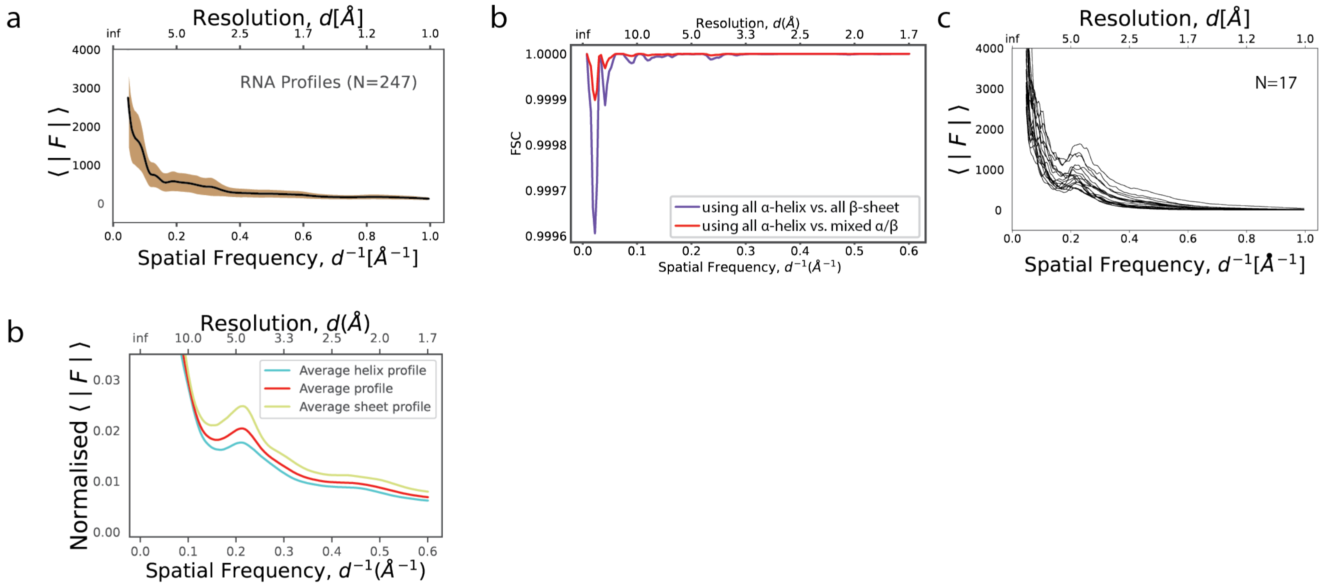

Fig. S5 (a) Average radial profiles for pure RNA structures. Solid line and shaded regions represent the mean and $\pm 1.0\ \sigma$ confidence intervals. (b) FSC curves between scaled maps obtained using an average α-profile or average β-profile (purple), and average α-profile or mixed α/β-profile (red). (c) Radial profile of 17 PDB structures showing convergence near the Wilson range (d) Combined plot showing the average α-profile, β-profile and a mixed α/β-profile.
